# Understanding How Microorganisms Respond to Acid pH Is Central to Their Control and Successful Exploitation

**DOI:** 10.3389/fmicb.2020.556140

**Published:** 2020-09-24

**Authors:** Peter A. Lund, Daniela De Biase, Oded Liran, Ott Scheler, Nuno Pereira Mira, Zeynep Cetecioglu, Estefanía Noriega Fernández, Sara Bover-Cid, Rebecca Hall, Michael Sauer, Conor O’Byrne

**Affiliations:** ^1^Institute of Microbiology and Infection, School of Biosciences, University of Birmingham, Birmingham, United Kingdom; ^2^Department of Medico-Surgical Sciences and Biotechnologies, Laboratory affiliated to the Istituto Pasteur Italia – Fondazione Cenci Bolognetti, Sapienza University of Rome, Latina, Italy; ^3^Department of Plant Sciences, MIGAL – Galilee Research Institute, Kiryat-Shemona, Israel; ^4^Department of Chemistry and Biotechnology, Tallinn University of Technology, Tallinn, Estonia; ^5^Institute for Bioengineering and Biosciences, Instituto Superior Técnico, Universidade de Lisboa, Lisbon, Portugal; ^6^Department of Chemical Engineering, KTH Royal Institute of Technology, Stockholm, Sweden; ^7^Department of Processing Technology, Nofima AS, Stavanger, Norway; ^8^IRTA, Food Safety Programme, Finca Camps i Armet, Monells, Spain; ^9^School of Biosciences, Kent Fungal Group, University of Kent, Canterbury, United Kingdom; ^10^Department of Biotechnology, University of Natural Resources and Life Sciences (BOKU), Vienna, Austria; ^11^Bacterial Stress Response Group, Microbiology, School of Natural Sciences, NUI Galway, Galway, Ireland

**Keywords:** acid stress, organic acids, intracellular pH homeostasis, industrial processes, food spoilage, photosynthesis, microbial infections

## Abstract

Microbes from the three domains of life, *Bacteria*, *Archaea*, and *Eukarya*, share the need to sense and respond to changes in the external and internal concentrations of protons. When the proton concentration is high, acidic conditions prevail and cells must respond appropriately to ensure that macromolecules and metabolic processes are sufficiently protected to sustain life. While, we have learned much in recent decades about the mechanisms that microbes use to cope with acid, including the unique challenges presented by organic acids, there is still much to be gained from developing a deeper understanding of the effects and responses to acid in microbes. In this perspective article, we survey the key molecular mechanisms known to be important for microbial survival during acid stress and discuss how this knowledge might be relevant to microbe-based applications and processes that are consequential for humans. We discuss the research approaches that have been taken to investigate the problem and highlight promising new avenues. We discuss the influence of acid on pathogens during the course of infections and highlight the potential of using organic acids in treatments for some types of infection. We explore the influence of acid stress on photosynthetic microbes, and on biotechnological and industrial processes, including those needed to produce organic acids. We highlight the importance of understanding acid stress in controlling spoilage and pathogenic microbes in the food chain. Finally, we invite colleagues with an interest in microbial responses to low pH to participate in the EU-funded COST Action network called EuroMicropH and contribute to a comprehensive database of literature on this topic that we are making publicly available.

## Introduction

**Figure 1:**
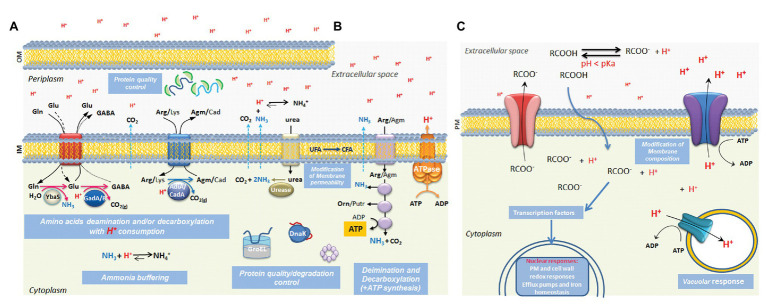
Summary of known mechanisms for responding to low pH stress in microbes. The schematic illustrates some of the better-studied mechanisms used by Gram negative **(A)** and Gram-positive bacteria **(B)** and yeasts **(C)** for responding to acidic conditions. Protonated weak organic acid permeation across the plasma membrane is illustrated in part **(C)** but this also applies in the inner membrane of bacterial cells. The details are discussed in the text where the relevant references are provided. OM, outer membrane; IM, inner membrane; PM: plasma membrane; Gln, glutamine; Glu, glutamate; GABA, γ-aminobutyric acid; Arg, arginine; Agm, agmatine; Lys, lysine; Cad, cadaverine; UFA, unsaturated fatty acids; CFA, cyclopropane fatty acids; RCOOH, organic acid in the undissociated form; RCOO^–^ , organic acid in the dissociated form (i.e., proton loss).

Microbes have successfully colonized almost every niche on Earth where they can access liquid water. Their survival and reproduction depend on their ability to produce appropriate responses to their immediate environmental conditions. One of the most significant environmental parameters impacting on growth and survival is the local concentration of protons (hydrogen ions, H^+^), which we measure as pH. Particular challenges arise for microbes when the proton concentration is high (acidic or low pH), which can occur through natural geochemical processes or through microbial metabolic processes that often generate organic acid by-products through redox balancing reactions. At low pH, the protonation of biological molecules influences their charge and therefore both structure and function can be adversely affected. Lipid bilayers are typically very impermeable to protons and this feature allows the proton gradient across a membrane to be utilized for energy generation (Mitchell, 1961). However weak organic acids, such as lactate or acetate, that are protonated at low pH (depending on the pK_a_ of their acidic groups) and therefore uncharged and more lipophilic, can permeate the lipid bilayer and release their protons in the intracellular environment where the pH is often closer to neutral and above the pK_a_ value of the acidic group (Figure 1). Thus, organic acids present a particular challenge for microbial cells as they can trigger cytoplasmic acidification and collapse proton gradients, especially when the extracellular milieu is acidic (Hirshfield et al., 2003; Mira and Teixeira, 2013).

Microbes typically respond to acid stress by preventing a damaging drop in intracellular pH (pH_i_) below a threshold level necessary for viability. Broadly, three distinct strategies are used to prevent such a critical drop in pH_i_ (Foster 2004; Krulwich et al., 2011; Lund et al., 2014). First, cells often employ enzyme-catalyzed reactions that consume protons: decarboxylation reactions often serve this purpose since a proton is irreversibly incorporated into the reaction product following the removal of CO_2_. Examples are the decarboxylation of amino acids, such as glutamate, arginine, or lysine, highlighted in Figure 1. Second, cells can deploy reactions that produce basic compounds to help neutralize the low pH. The production of ammonia from urea or amine-containing amino acids such as arginine or glutamine is commonly used to counteract acidity (Krulwich et al., 2011; Pennacchietti et al., 2018). Third, many microbial cell types eliminate protons from the cells at the expense of ATP consumption. Protons can be effluxed from some bacteria using the F_1_Fo-ATPase (Russell, 2007; Krulwich et al., 2011) while in some fungal or yeast species, a dedicated proton translocating efflux pump (in *Saccharomyces cerevisiae* it is the well-studied Pma1 proton pump) is used (Mira et al., 2010). The anions of organic acids can themselves be inhibitory when they accumulate at high intracellular concentrations and both bacteria and yeasts have evolved mechanisms to efflux the anions *via* membrane pumps (Mira et al., 2010; Du et al., 2018).

In addition to these mechanisms for maintaining pH_i_ cells often deploy specific protective systems that help cope with acid stress. Modification of the lipid composition of the cytoplasmic membrane to reduce the permeability to protons is found in many microbes. In some bacteria, cyclopropane fatty acids are produced at higher levels under acid stress and serve to protect against acid pH by decreasing membrane permeability to protons (Chang and Cronan 1999; Shabala and Ross 2008). Specific acid-stress induced systems to chaperone and refold proteins damaged by the low pH are often employed to maintain critical enzyme-based functions in the cell (Hong et al., 2012; Voth and Jakob, 2017). Damage to the genetic material by acid pH or organic acids, often caused indirectly by generating reactive oxygen species (Schellhorn and Stones, 1992; Kim et al., 2006; Bruno-Bárcena et al., 2010), can trigger a DNA damage response in both bacteria and yeast (Ribeiro et al., 2006; Jeong et al., 2008).

Acid is encountered by microbes in many environments, such as certain soil types, acidic lakes and mines, geothermal sites, decomposing organic matter, as well in a variety of human-associated niches, including acidic food products, industrial fermentations, and waste-treatment facilities and in the gastrointestinal tract and other anatomical sites during infections. Microorganisms from widely different phylogenetic groups have evolved to occupy many of these niches, including acidophilic bacterial and archaeal species that have growth optima as low as pH1–2 (Baker-Austin and Dopson, 2007). In most cases, efficient pH_i_ homeostasis as described above is the principle means used to survive in extreme acid environments, although in some cases cytoplasmic proteins can evolve to function well at low pH, as has been found in the acetic acid producing bacterium *Acetobacter aceti* (Francois and Kappock, 2007). Here, we examine some of the key areas where a better understanding of the responses to acid, and the stresses associated with acidification, could help with the control of pathogenic or spoilage microorganisms and help to optimize the functionality of microbes being used to produce commercially useful end products, contributing to the development of a global circular economy, where reuse of waste streams is prioritized to maximize long-term sustainability.

## Experimental Approaches to Study Low pH Responses

**Figure 2:**
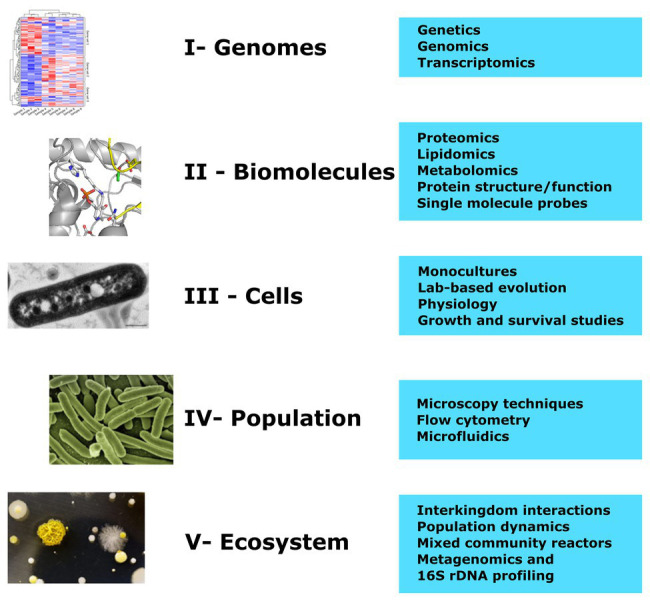
Approaches to understanding the responses of microbes to low pH at different organizational levels. The graphic highlights the research approaches (blue boxes) that can be used to study acid pH responses at each organizational level, from genomes through to ecosystems (represented by the images on the left). The presence of specific genes in the genome and their regulation in response to acid determines the cells’ capacity to cope with low pH stress (I). Acid-induced proteins are responsible for structural and metabolic changes in the cell, resulting in altered molecular composition at low pH (II). At the cellular level, acidic pH can have strong effect on the overall growth and behavior of the microbes (III), but with a population of cells there can be genetic and biochemical heterogeneity leading to different behavioral outcomes (IV). Within mixed communities both the population structure and inter-species and intra-species interactions can be strongly influenced by changes in pH (V).

Low pH has effects at a variety of biological levels, including genomic, biochemical, cellular, population, and ecosystem levels. Distinct research approaches are needed to develop a full understanding of how each level is impacted by low pH (Figure 2). (I) High-throughput genomic approaches combined with modeling have enabled the identification of key genes, regulators, and pathways responsible for acid responses (for example, Stincone et al., 2011; Chakraborty and Kenney, 2018; Du et al., 2019). (II) Intracellular changes of metabolites, including the actual levels of free protons can be observed directly, for example, *via* NMR (Chen et al., 2020) or indirectly using a range of different fluorescent probes (Slonczewski et al., 2009; Kenney, 2019; Joniak et al., 2020), and these measurements can be done at the population or single cell level. Similarly, changes in growth in response to different pH or different acids can be monitored at single cell level (III; Mitosch et al., 2017) or within a population as a whole (IV; Ratzke and Gore, 2017; Bushell et al., 2019). Within mixed microbial communities population dynamics can be strongly influenced by the changing environmental pH (Ratzke and Gore, 2017). This can impact the ecology of both natural (e.g., soil and aquatic) and anthropogenic environments such as those found in anaerobic digesters for wastewater treatment or in mixed inocula used for food fermentations. Specific tools, including metagenomics, 16S rRNA profiling, label-free meta-proteomics, spatiotemporal analytical techniques [e.g., fluorescence *in situ* hybridization (FISH)] are needed to investigate in more detail how population composition can be influenced by acid stress in complex microbial communities.

There are still significant gaps in understanding microbial responses to acidic pH that surely can be filled by using well known approaches from neighboring microbiology fields, or by many rapidly advancing novel technologies and approaches. For example, laboratory evolution experiments combined with whole genome sequencing have given new insights into acid resistance and tolerance (Harden et al., 2015; Creamer et al., 2017). Metagenomic studies can give information on many levels starting from species composition down to specific pathways and genes (Quatrini and Johnson, 2018). Linking genomics tools with phenotypical studies will definitely bring more insight into genetic mechanisms behind microbial responses to acidic environment (Guerreiro et al., 2020). Mathematical modeling and computational approaches will also play an important role in understanding microbial physiological processes during acid adaptation (Mancini et al., 2020) and interactions between bacteria (Ratzke and Gore, 2017). Microfluidics and increasingly sophisticated image and molecular analyses will assist with high-throughput single cell studies (Liew et al., 2019; Scheler et al., 2019).

## Low pH in Relation to Infectious Diseases

To successfully colonize the human host, microbes face a myriad of challenges that include acid stress. This is the case for pathogens colonizing acidic niches such as the stomach (pH≈1–2), the vaginal epithelium (pH≈4), or the colon (pH≈5.7; Fallingborg, 1999; O’Hanlon et al., 2019). Acidity is produced by distinct processes in these environments. HCl is made by gastric cells in the stomach, while in the vagina or in the intestine it results from weak organic acids produced by the commensal microbiota (Fallingborg, 1999; O’Hanlon et al., 2019). Pathogens entering the host *via* these sites, including food-borne pathogens like *Salmonella enterica* or *Listeria monocytogenes*, or the vaginal pathogen *Candida albicans*, typically have adaptive responses to acid stress, such as the alkalinization of the microenvironment through the production of buffering compounds (Merrell, 2002; Danhof et al., 2016), increased activity of proton pumps (Li et al., 2018), or the decarboxylation of glutamate or arginine (Lund et al., 2014). The ability to metabolize organic acids such as lactate is another important adaptive response, contributing to reduced accumulation of these molecules inside infecting cells while simultaneously improving growth in sites that are usually deprived of carbon (Childers et al., 2016; Louis and Flint, 2017). Low pH and short chain fatty acids (SCFA, also referred to as volatile fatty acids, VFA) have been found to modulate the efficiency of immune function in a variety of ways, including effects on the epithelial barrier, immune cell function, and cytokine production (Lardner, 2001; Corrêa-Oliveira et al., 2016). More recently, adaptation of microbes to acids has been shown to affect innate immune responses through modulation of the microbial cell surface, with weak organic acids promoting evasion of the innate immune system (Ballou et al., 2017) and strong acids eliciting a strong pro-inflammatory immune response (Sherrington et al., 2017). The regulatory mechanisms controlling these responses in the infecting species are often poorly characterized, especially in emerging pathogens or in less genetically and/or experimentally accessible species. The identification of these regulatory proteins/pathways could represent a novel cohort of possible therapeutic targets, particularly important with the global rise in antimicrobial resistance. Acid pH also modulates the interaction between pathogens and antimicrobials (Danby et al., 2012; Yang et al., 2017; Lourenço et al., 2019; Lu et al., 2020). The possibility of exploring molecules whose activity can be potentiated at low pH (e.g., organic acids) as treatments, either alone or in combination with antifungals/antibiotics, seems worth pursuing, with some preliminary results showing that SCFA at low pH can increase the efficacy of azoles and antibiotics (Yang et al., 2017; Li et al., 2018). The use of SCFAs to control microbial growth in food has been successfully exploited by the food/beverages industry for many years, and interest in translating their applications into a clinical setting is also growing (Nagoba et al., 2013). Infectious disease microbiology and food microbiology have traditionally been distinct fields and the extensive knowledge acquired in the latter may be of great value for clinicians if researchers from these groups fully explore the possibility of exploiting weak acids as a treatment for infections. The application of weak acids in situations where the infections are topical and amenable, such as in burns and in device-associated biofilms, has significant potential and deserves further study (Hughes and Webber, 2017).

## Low pH and Photosynthesis

The rise in partial pressure of CO_2_ in the atmosphere, due to rise in anthropogenic CO_2_ emissions, alters the chemistry of the oceans by decreasing pH rapidly (Ren et al., 2020). Since phytoplankton in the oceans are responsible for about 20% of the global generation of organic compounds through carbon assimilation and photosynthesis (Li et al., 2017) it is imperative to understand the implications of reduced pH on their metabolism and physiology. *Cyanobacteria* tolerate slightly acidic pH conditions (pH 5.7–6.0) during times of acid rain and ocean acidification (Ren et al., 2014), while acidophile protists tolerate pH as low as 1 and dwell in geothermal hot springs or acid mine drainage pools (Gross, 2000). In spite of low pH, these photosynthetic microbes are able to maintain a near neutral pH within their cytoplasm (Kallas and Castenholz, 1982; Giraldez-Ruiz et al., 1997; Schönknecht et al., 2013). *Cyanobacteria* exhibit both a carbon-concentrating mechanism coupled to proton extrusion and lipopolysaccharide synthesis mechanisms, which are upregulated during acidified conditions (Katoh et al., 1996; Matsuhashi et al., 2015). The Ca^+2^ cation has also been shown to promote acid tolerance by decreasing porosity of the cytoplasmic membrane (Giraldez-Ruiz et al., 1999). There is evidence that photosynthetic acidophiles gain tolerance to low pH *via* horizontal gene transfer from other protists in their vicinity (Olsson et al., 2017). In addition to the burden of pH homeostasis, these microbes are also exposed to increased salt and heavy metal stress, due to increased solubility of these compounds in acidic conditions (Spijkerman, 2007). Acidophilic protists cope with pH homeostasis on top of increased salt and heavy metal stress by upregulating heat shock proteins as well as ATP-H^+^ pumps (Gerloff-Elias et al., 2006).

## Acid in Industrial Processes

Microbial responses to low pH are of particular importance for the production of organic acids, which are industrially useful compounds that are produced at high volume, but often with low value per kilogram. Maintaining the culture pH as low as possible helps to simplify the purification of the acid considerably, and thereby lowers the production costs (Sauer et al., 2008). Lactic acid production is one example. It is used extensively as a food additive and can also be used as a precursor in the production of biodegradable polyesters. Lactic acid bacteria (LAB) are the best natural producers but they have the major disadvantage that their growth decreases at pH values lower than 5, but a pH value as low as 3 is desirable for purification. Many industrial approaches focus therefore on engineered yeasts such as Baker’s yeast or *Pichia kudriavzevii*, which are typically much more acid tolerant than LAB (Sun et al., 2020). Another example is microbial production of succinic acid. Very different cell factories have been developed for its production (among them the prokaryotes *Mannheimia succiniciproducens*, *Escherichia coli*, *Basfia succiniciproducens*, *Actinobacillus succinogenes*, and *Corynebacterium glutamicum* and yeasts, including *Saccharomyces cerevisiae*, *Pichia kudriavzevii*, and *Yarrowia lipolytica*). However, in the end yeasts turned out to be industrially more successful due to their low pH tolerance (Ahn et al., 2016; Franco-Duarte et al., 2017). Low pH tolerance is also known to be an important factor in the successful production of acetic acid (Sauer et al., 2008). Differences in low pH tolerance are also exploited industrially to favor desired production organisms over contaminants, when the processes are performed under non-sterile conditions. For example, yeast cells used in bio-ethanol production are washed with sulfuric acid to reduce bacterial contaminants, allowing the biomass to be re-used and keeping undesired contaminating microorganisms in check (Basso et al., 2011).

Environmental biotechnology has emerged as an important field aimed at establishing sustainable processes that seek to exploit the potential of human-generated waste streams, thereby contributing to the development of a circular economy. For example, volatile fatty acids (VFA) can be produced by mixed culture fermentation of waste streams under low pH conditions (Atasoy et al., 2018). This approach relies on mixed communities, frequently comprising an undefined consortium of microbes, thus avoiding the necessity for highly controlled working conditions but requiring tight control of the reactor pH to prevent acidification. While reactor stability during continuous VFA production can be a problem, lower pH conditions can help to stabilize it during long-term operation (Bengtsson et al., 2008; Zhou et al., 2018; Owusu-Agyeman et al., 2020). Inoculum, reactor type, waste stream composition as well as pH can all affect the reactor stability and therefore the amount and type of end-product (Lee et al., 2014; Domingos et al., 2017). Moreover, problems in reactor stability can make the scale-up process from pilot to full-scale challenging for wastewater treatment plants. Therefore, a robust pH control in VFA production to maintain low pH conditions is essential for continuous reactor operation. For some applications, maintaining the reactor at a low pH could provide the necessary selection pressure to stabilize the microbial community and improve the efficiency at full scale (Esteban-Gutiérrez et al., 2018).

## Low pH in Food and Beverages

Acidity is an inherent property of many foods, including citrus fruits, berries, and honey, and can also be caused by fermentation by endogenous bacteria or added starter cultures (Pérez-Díaz et al., 2017). Acidity in food acts as a hurdle to the growth of spoilage and pathogenic microbes, especially when combined with other hurdles such as low water activity or low temperature, and can greatly improve food shelf life and safety (Leistner and Gorris, 1995). Indeed many organic acids, including acetic, citric, formic, lactic, propionic, sorbic, and benzoic acids, are routinely used in food products as acidity regulators, flavor enhancers, and antioxidants and because of their broad spectrum antimicrobial activity (Theron and Lues, 2011; Anyasi et al., 2017). The bacteriostatic or bactericidal properties of organic acids depend on the physiological status of the cells and the physicochemical properties of the surrounding environment, and in particular the extent of dissociation of the acid (Ricke, 2003; Lianou et al., 2012). However, several spoilage bacteria, yeasts, and molds have developed resistance to organic acids. Two examples are *Alicyclobacillus acidoterrestris*, a major spoilage bacterium in fruit juice (Pornpukdeewattana et al., 2020), and *Aspergillus niger*, a fungus that can decarboxylate sorbic acid and consequently facilitate the growth of other more acid sensitive microorganisms (Stratford et al., 2012). Additionally, there is some evidence that acid stress responses can induce cross-protection against subsequent hurdles during processing, such as heat and osmotic stress, and this has the potential to compromise food safety (Yousef and Juneja, 2003; Beales, 2004; Kim et al., 2019).

Quantitative microbial ecology, also known as predictive microbiology, uses mathematical algorithms to better predict the growth response of the microorganisms to intrinsic and extrinsic factors, including pH (McMeekin and Ross, 2002), and this is an important approach in predicting food safety. A number of tools are already available for this (Tenenhaus-Aziza and Ellouze, 2015), with pH being an input factor in almost all the models; the concentration of organic acids is also considered in some of the tools. When properly validated, predictive models constitute key tools for developing quantitative microbial risk assessments (QMRA), addressing public health issues (food-borne intoxications and infections), and food production and preservation challenges, aimed at minimizing food spoilage and protecting the food against food-borne pathogens. However, the predictive performance of these tools has not always been proven in real food systems and in some cases, further research is needed before an accurate prediction of the microbial growth can be achieved (Martinez-Rios et al., 2019). A better understanding of how poorly studied relevant microorganisms respond to acidity in foods would be invaluable in further strengthening these predictive models.

## Future Perspectives and Opportunities

This brief review has highlighted a few of the many ways, in which the deeper understanding of microbial responses to low pH present both a compelling scientific challenge, and an opportunity to make significant advances in fields as diverse as bioprocessing, environmental, food and beverage, and clinical microbiology. In the latter two cases, the drive is often to kill or prevent the growth of undesirable organisms, whereas in the former it is often to achieve better growth, but these are two sides of the same coin: an improved understanding of microbial responses to acid stress has the potential to enhance our ability to do both. We believe that improving our understanding in these areas by establishing closer links between workers in all these diverse fields, some of which do not currently overlap significantly. Establishing cross-disciplinary collaborations between researchers with an interest in the low pH responses of phylogenetically distinct groups of microorganisms and researchers who employ different experimental approaches, is a major goal of the EU-funded COST Action project EuroMicropH, on which the authors of this perspective article collaborate. As part of this project, we are building a repository of scientific literature that will be publicly available that describes every aspect of how microbes, across all domains of life, sense and respond to acid stress. We have an open call to all researchers with an interest in this field to send us research articles, reviews or indeed any documents that might shed light on any fundamental aspects of the problem in any microbial group. Full details of how to submit such articles for inclusion in the repository, and about future activities of our project and how to get involved with it, are available on the EuroMicropH website^1^.

## Author’s Note

All authors are affiliated to the EuroMicropH COST project (COST Action CA18113). https://euromicroph.eu/.

## Author Contributions

All authors contributed to researching, writing, and editing of this perspective article. All authors contributed to the article and approved the submitted version.

### Disclaimer

EF is currently employed with the European Food Safety Authority (EFSA) at the Nutrition Unit that provides scientific and administrative support to the NDA panel in the area of safety assessment of novel foods. However, the present article is published under the sole responsibility of the author/s and may not be considered as an EFSA scientific output. The positions and opinions presented in this article are those of the author/s alone and are not intended to represent the views/any official position or scientific works of EFSA. To know about the views or scientific outputs of EFSA, please consult its website under http://www.efsa.europa.eu.

### Conflict of Interest

The authors declare that the research was conducted in the absence of any commercial or financial relationships that could be construed as a potential conflict of interest.

## Footnotes

^1^www.euromicroph.eu
